# 4,4′-(Ethane-1,2-diyl)dipyridinium bis­(2-hy­droxy­benzoate)

**DOI:** 10.1107/S160053681003816X

**Published:** 2010-10-02

**Authors:** Shie Fu Lush, Fwu Ming Shen

**Affiliations:** a General Education Center, Yuanpei University, No. 306, Yuanpei St, HsinChu 30015, Taiwan; b Department of Biotechnology, Yuanpei University, No. 306, Yuanpei St, HsinChu 30015, Taiwan

## Abstract

In the crystal structure of the title compound, C_12_H_14_N_2_^2+^·2C_7_H_5_O_3_^−^, the cations and anions are linked *via* N—H⋯O hydrogen bonds and weak inter­molecular C—H⋯O inter­actions also occur. π–π stacking is observed between the nearly parallel benzene and pyridine rings [dihedral angle = 6.03 (8)°], the centroid–centroid separation being 3.7546 (16) Å. The 4,4′-(ethane-1,2-diyl)dipyridinium cation is centrosymmetric and the mid-point of the ethyl­ene C—C bond is located on an inversion center. An intra­molecular O—H⋯O hydrogen bond occurs in the anion.

## Related literature

For the structure of 4,4′-(ethane-1,2-diyl)dipyridinium bis­(3,5-dinitro­benzoate), see: Burchell *et al.* (2001 ▶). For the structure of 4,4′-(ethane-1,2-diyl)dipyridinium bis­(hydrogen maleate), see: Bowes *et al.* (2003 ▶). For deprotonated salicylic acid, see: Chitradevi *et al.* (2009 ▶); Fun *et al.* (2010 ▶); Quah *et al.* (2010 ▶).
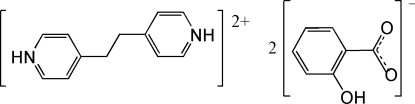


## Experimental

### 

#### Crystal data

C_12_H_14_N_2_^2+^·2C_7_H_5_O_3_^−^
*M* *_r_* = 460.47
Monoclinic, 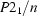

*a* = 8.622 (3) Å
*b* = 6.867 (2) Å
*c* = 19.566 (6) Å
β = 101.324 (6)°
*V* = 1135.9 (6) Å^3^
*Z* = 2
Mo *K*α radiation
μ = 0.10 mm^−1^
*T* = 297 K
0.42 × 0.26 × 0.17 mm

#### Data collection

Bruker SMART CCD area-detector diffractometer
6165 measured reflections
2246 independent reflections
1645 reflections with *I* > 2σ(*I*)
*R*_int_ = 0.053

#### Refinement

*R*[*F* ^2^ > 2σ(*F* ^2^)] = 0.047
*wR*(*F* ^2^) = 0.147
*S* = 1.05
2246 reflections
155 parameters
1 restraint
H-atom parameters constrained
Δρ_max_ = 0.21 e Å^−3^
Δρ_min_ = −0.25 e Å^−3^

### 

Data collection: *SMART* (Bruker, 2000 ▶); cell refinement: *SAINT* (Bruker, 1999 ▶); data reduction: *SAINT*; program(s) used to solve structure: *SHELXS97* (Sheldrick, 2008 ▶); program(s) used to refine structure: *SHELXL97* (Sheldrick, 2008 ▶); molecular graphics: *ORTEP-3* (Farrugia, 1997 ▶); software used to prepare material for publication: *PLATON* (Spek, 2009 ▶).

## Supplementary Material

Crystal structure: contains datablocks global, I. DOI: 10.1107/S160053681003816X/xu5033sup1.cif

Structure factors: contains datablocks I. DOI: 10.1107/S160053681003816X/xu5033Isup2.hkl

Additional supplementary materials:  crystallographic information; 3D view; checkCIF report

## Figures and Tables

**Table 1:** Hydrogen-bond geometry (Å, °)

*D*—H⋯*A*	*D*—H	H⋯*A*	*D*⋯*A*	*D*—H⋯*A*
N—H1*A*⋯O1	0.86	1.70	2.556 (2)	177
O3—H3*A*⋯O2	0.82	1.76	2.545 (2)	160
C11—H11⋯O3^i^	0.93	2.55	3.406 (3)	154

Symmetry code: (i) 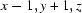.

**Fig. 1.**
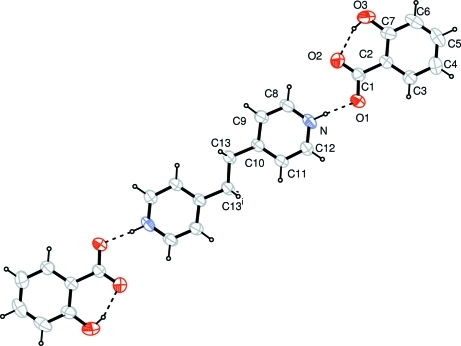
The structure of the title compound with the atom numbering scheme. Displacement ellipsoids for non-H atoms are drawn at the 50% probability level. Dashed lines indicate hydrogen bonding [symmetry code: (i) -x, 2 - y, 1 - z].

## supplementary crystallographic information

### Comment

There are numerous examples of saliclyic acid compounds in which the salicylic acid act as deprotonated anions (Quah *et al.*, 2010; Fun *et al.*, 2010; Chitradevi *et al.*, 2009). Some 4,4-ethylenedipyridinium salts have also reported previously (Burchell *et al.* 2001; Bowes *et al.* 2003).

The crystal structure of the title proton-transfer compound of salicylic acid with 4,4'-(ethane-1,2-diyl)dipyridine consists of 4,4'-(ethane-1,2-diyl)dipyridinium cations and 2-hydroxybenzoate anionst (Fig. 1). The 4,4'-(ethane-1,2-diyl)dipyridinium cation is centro-symmetric, with the mid-point of ethylene C—C bond located on the inversion center. Two salicylate anions have intramolecular hydrogen bonding. The 4,4'-(ethane-1,2-diyl)dipyridinium cation is linked by N—H···O hydrogen bond to adjacent salicylate anions.

Intermolecular weak C—H···O hydrogen bonding is present in the crystal structure (Table 1). On the other hand, π–π ring stacking is also observed, the centroid–centroid separation between the benzene and pyridine ring, *Cg*1(N/C8—C12)··· *Cg*2^iii^(C2—C7), is 3.7546 (16) Å and dihedral angle between two rings is 6.03 (8)° [symmetry code: (iii) = *x*, 1 + *y*, *z*].

### Experimental

The salicylic acid (138.0 mg, 1.0 mmol) and 4,4'-(ethane-1,2-diyl)dipyridine (184 mg, 1.0 mmol) were dissolved in 20 ml me thanol-water (1:1), the solution was refluxed for 30 min. The filtered solution was transferred to a 25 ml tube, after one week at room temperature colorless transparent crystals formed (yield 56.78%).

### Refinement

H atoms bonded to O and N atoms were located in a difference Fourier map and refined with the distances constraints of O—H = 0.82, N—H = 0.86 Å, and *U*_iso_(H) = 1.2*U*_eq_(N) and 1.5*U*_eq_(O). Other H atoms were positioned geometrically with C—H = 0.93 (aromatic) and 0.97 Å (methylene), and were refined using a riding model with *U*_iso_(H) = 1.2*U*_eq_(C).

### Crystal data

**Table:**

C_12_H_14_N_2_^2+^·2C_7_H_5_O_3_^−^	*F*(000) = 484
*M**_r_* = 460.47	*D*_x_ = 1.346 Mg m^−^^3^
Monoclinic, *P*2_1_/*n*	Mo *K*α radiation, λ = 0.71073 Å
Hall symbol: -P 2yn	Cell parameters from 2343 reflections
*a* = 8.622 (3) Å	θ = 3.6–25.9°
*b* = 6.867 (2) Å	µ = 0.10 mm^−^^1^
*c* = 19.566 (6) Å	*T* = 297 K
β = 101.324 (6)°	Prism, colorless
*V* = 1135.9 (6) Å^3^	0.42 × 0.26 × 0.17 mm
*Z* = 2	

### Data collection

**Table:**

Bruker SMART CCD area-detector diffractometer	1645 reflections with *I* > 2σ(*I*)
Radiation source: fine-focus sealed tube	*R*_int_ = 0.053
graphite	θ_max_ = 26.1°, θ_min_ = 2.1°
Detector resolution: 9 pixels mm^-1^	*h* = −5→10
ω scan	*k* = −8→8
6165 measured reflections	*l* = −24→23
2246 independent reflections	

### Refinement

**Table:**

Refinement on *F*^2^	Secondary atom site location: difference Fourier map
Least-squares matrix: full	Hydrogen site location: inferred from neighbouring sites
*R*[*F*^2^ > 2σ(*F*^2^)] = 0.047	H-atom parameters constrained
*wR*(*F*^2^) = 0.147	*w* = 1/[σ^2^(*F*_o_^2^) + (0.0761*P*)^2^ + 0.1342*P*] where *P* = (*F*_o_^2^ + 2*F*_c_^2^)/3
*S* = 1.05	(Δ/σ)_max_ < 0.001
2246 reflections	Δρ_max_ = 0.21 e Å^−^^3^
155 parameters	Δρ_min_ = −0.25 e Å^−^^3^
1 restraint	Extinction correction: *SHELXL97* (Sheldrick, 2008), Fc^*^=KFc[1+0.001Fc^2^λ^3^/sin(2Θ)]^-1/4^
Primary atom site location: structure-invariant direct methods	Extinction coefficient: 0.015 (3)

### Special details

**Table:**

Geometry. Bond distances, angles *etc*. have been calculated using the rounded fractional coordinates. All su's are estimated from the variances of the (full) variance-covariance matrix. The cell e.s.d.'s are taken into account in the estimation of distances, angles and torsion angles
Refinement. Refinement on *F*^2^ for ALL reflections except those flagged by the user for potential systematic errors. Weighted *R*-factors *wR* and all goodnesses of fit *S* are based on *F*^2^, conventional *R*-factors *R* are based on *F*, with *F* set to zero for negative *F*^2^. The observed criterion of *F*^2^ > σ(*F*^2^) is used only for calculating -*R*-factor-obs *etc*. and is not relevant to the choice of reflections for refinement. *R*-factors based on *F*^2^ are statistically about twice as large as those based on *F*, and *R*-factors based on ALL data will be even larger.

### Fractional atomic coordinates and isotropic or equivalent isotropic displacement parameters (Å^2^)

**Table:**

	*x*	*y*	*z*	*U*_iso_*/*U*_eq_	
N	0.23112 (16)	0.41868 (19)	0.59247 (7)	0.0507 (5)	
C8	0.30204 (19)	0.4936 (2)	0.54421 (9)	0.0508 (6)	
C9	0.25306 (19)	0.6652 (2)	0.51103 (9)	0.0502 (5)	
C10	0.12421 (17)	0.7649 (2)	0.52708 (8)	0.0419 (5)	
C11	0.05191 (19)	0.6837 (2)	0.57748 (9)	0.0504 (5)	
C12	0.1084 (2)	0.5109 (3)	0.60879 (10)	0.0545 (6)	
C13	0.07080 (18)	0.9519 (2)	0.48984 (9)	0.0483 (5)	
O1	0.32265 (15)	0.10150 (17)	0.65611 (7)	0.0612 (5)	
O2	0.48925 (16)	0.07561 (19)	0.58403 (7)	0.0689 (5)	
O3	0.67244 (17)	−0.2176 (2)	0.59167 (8)	0.0822 (6)	
C1	0.43333 (19)	0.0129 (2)	0.63306 (9)	0.0463 (5)	
C2	0.48956 (17)	−0.1735 (2)	0.66799 (8)	0.0425 (5)	
C3	0.4252 (2)	−0.2478 (3)	0.72231 (9)	0.0515 (6)	
C4	0.4772 (2)	−0.4221 (3)	0.75408 (10)	0.0660 (7)	
C5	0.5946 (2)	−0.5245 (3)	0.73117 (11)	0.0696 (7)	
C6	0.6594 (2)	−0.4550 (3)	0.67733 (12)	0.0670 (7)	
C7	0.6078 (2)	−0.2797 (2)	0.64510 (10)	0.0515 (6)	
H1A	0.26440	0.31150	0.61290	0.0610*	
H8	0.38750	0.42790	0.53250	0.0610*	
H9	0.30600	0.71500	0.47780	0.0600*	
H11	−0.03430	0.74520	0.59020	0.0600*	
H12	0.05870	0.45740	0.64250	0.0650*	
H13A	0.04610	0.92600	0.44020	0.0580*	
H13B	0.15830	1.04320	0.49830	0.0580*	
H3	0.34550	−0.17890	0.73760	0.0620*	
H3A	0.62520	−0.11430	0.58230	0.1230*	
H4	0.43350	−0.46980	0.79060	0.0790*	
H5	0.63030	−0.64170	0.75240	0.0830*	
H6	0.73840	−0.52550	0.66230	0.0800*	

### Atomic displacement parameters (Å^2^)

**Table:**

	*U*^11^	*U*^22^	*U*^33^	*U*^12^	*U*^13^	*U*^23^
N	0.0485 (8)	0.0343 (7)	0.0662 (9)	0.0103 (6)	0.0038 (7)	0.0061 (6)
C8	0.0438 (9)	0.0412 (9)	0.0677 (11)	0.0099 (7)	0.0118 (8)	0.0010 (8)
C9	0.0452 (9)	0.0448 (9)	0.0623 (10)	0.0069 (7)	0.0146 (7)	0.0043 (8)
C10	0.0356 (8)	0.0358 (8)	0.0531 (9)	0.0032 (6)	0.0055 (6)	0.0019 (7)
C11	0.0441 (9)	0.0454 (9)	0.0637 (10)	0.0137 (7)	0.0154 (8)	0.0095 (8)
C12	0.0527 (10)	0.0467 (9)	0.0660 (11)	0.0106 (8)	0.0166 (8)	0.0140 (8)
C13	0.0451 (9)	0.0402 (9)	0.0606 (10)	0.0084 (7)	0.0128 (7)	0.0093 (8)
O1	0.0669 (8)	0.0460 (7)	0.0773 (9)	0.0201 (6)	0.0307 (6)	0.0110 (6)
O2	0.0834 (10)	0.0534 (8)	0.0801 (9)	0.0133 (7)	0.0407 (8)	0.0186 (7)
O3	0.0732 (9)	0.0799 (10)	0.1084 (12)	0.0229 (8)	0.0541 (9)	0.0127 (9)
C1	0.0490 (9)	0.0376 (8)	0.0532 (10)	0.0033 (7)	0.0126 (7)	0.0005 (7)
C2	0.0406 (8)	0.0372 (8)	0.0480 (9)	0.0030 (6)	0.0043 (6)	−0.0019 (7)
C3	0.0542 (10)	0.0501 (10)	0.0505 (9)	0.0075 (8)	0.0108 (8)	0.0028 (8)
C4	0.0771 (13)	0.0583 (11)	0.0598 (11)	0.0060 (10)	0.0067 (9)	0.0159 (9)
C5	0.0691 (12)	0.0474 (10)	0.0823 (14)	0.0118 (10)	−0.0094 (10)	0.0130 (10)
C6	0.0499 (11)	0.0530 (11)	0.0933 (15)	0.0185 (9)	0.0026 (10)	−0.0063 (11)
C7	0.0415 (9)	0.0474 (10)	0.0657 (11)	0.0045 (7)	0.0111 (8)	−0.0046 (8)

### Geometric parameters (Å, °)

**Table:**

O1—C1	1.286 (2)	C11—H11	0.9300
O2—C1	1.233 (2)	C12—H12	0.9300
O3—C7	1.347 (2)	C13—H13A	0.9700
O3—H3A	0.8200	C13—H13B	0.9700
N—C8	1.326 (2)	C1—C2	1.487 (2)
N—C12	1.325 (2)	C2—C7	1.397 (2)
N—H1A	0.8600	C2—C3	1.389 (2)
C8—C9	1.371 (2)	C3—C4	1.382 (3)
C9—C10	1.392 (2)	C4—C5	1.378 (3)
C10—C11	1.383 (2)	C5—C6	1.371 (3)
C10—C13	1.503 (2)	C6—C7	1.391 (3)
C11—C12	1.380 (3)	C3—H3	0.9300
C13—C13^i^	1.509 (2)	C4—H4	0.9300
C8—H8	0.9300	C5—H5	0.9300
C9—H9	0.9300	C6—H6	0.9300
			
C7—O3—H3A	101.00	C13^i^—C13—H13B	108.00
C8—N—C12	119.22 (15)	C13^i^—C13—H13A	108.00
C8—N—H1A	120.00	O1—C1—C2	116.34 (14)
C12—N—H1A	120.00	O2—C1—C2	121.09 (15)
N—C8—C9	121.90 (15)	O1—C1—O2	122.57 (14)
C8—C9—C10	120.06 (15)	C1—C2—C7	119.65 (14)
C9—C10—C13	119.52 (14)	C3—C2—C7	118.68 (15)
C11—C10—C13	123.49 (14)	C1—C2—C3	121.65 (15)
C9—C10—C11	116.99 (13)	C2—C3—C4	121.27 (16)
C10—C11—C12	119.61 (15)	C3—C4—C5	119.33 (18)
N—C12—C11	122.22 (17)	C4—C5—C6	120.56 (19)
C10—C13—C13^i^	115.66 (13)	C5—C6—C7	120.48 (18)
N—C8—H8	119.00	O3—C7—C6	118.79 (16)
C9—C8—H8	119.00	C2—C7—C6	119.68 (16)
C8—C9—H9	120.00	O3—C7—C2	121.52 (14)
C10—C9—H9	120.00	C2—C3—H3	119.00
C12—C11—H11	120.00	C4—C3—H3	119.00
C10—C11—H11	120.00	C3—C4—H4	120.00
N—C12—H12	119.00	C5—C4—H4	120.00
C11—C12—H12	119.00	C4—C5—H5	120.00
C10—C13—H13B	108.00	C6—C5—H5	120.00
H13A—C13—H13B	107.00	C5—C6—H6	120.00
C10—C13—H13A	108.00	C7—C6—H6	120.00
			
C12—N—C8—C9	0.7 (2)	O2—C1—C2—C3	−178.62 (16)
C8—N—C12—C11	−0.4 (3)	O2—C1—C2—C7	0.1 (2)
N—C8—C9—C10	−0.8 (3)	C1—C2—C3—C4	179.41 (16)
C8—C9—C10—C11	0.6 (2)	C7—C2—C3—C4	0.7 (3)
C8—C9—C10—C13	−179.43 (15)	C1—C2—C7—O3	−0.2 (2)
C9—C10—C11—C12	−0.4 (2)	C1—C2—C7—C6	−179.40 (16)
C13—C10—C11—C12	179.70 (16)	C3—C2—C7—O3	178.52 (16)
C9—C10—C13—C13^i^	179.34 (14)	C3—C2—C7—C6	−0.7 (3)
C11—C10—C13—C13^i^	−0.7 (2)	C2—C3—C4—C5	−0.3 (3)
C10—C11—C12—N	0.2 (3)	C3—C4—C5—C6	−0.1 (3)
C10—C13—C13^i^—C10^i^	−179.97 (17)	C4—C5—C6—C7	0.1 (3)
O1—C1—C2—C3	0.3 (2)	C5—C6—C7—O3	−178.96 (18)
O1—C1—C2—C7	178.98 (15)	C5—C6—C7—C2	0.3 (3)

Symmetry codes: (i) −*x*, −*y*+2, −*z*+1.

### Hydrogen-bond geometry (Å, °)

**Table:**

*D*—H···*A*	*D*—H	H···*A*	*D*···*A*	*D*—H···*A*
N—H1A···O1	0.86	1.70	2.556 (2)	177
O3—H3A···O2	0.82	1.76	2.545 (2)	160
C11—H11···O3^ii^	0.93	2.55	3.406 (3)	154

Symmetry codes: (ii) *x*−1, *y*+1, *z*.
